# Usefulness of point-of-care ultrasound for rapid assessment of sarcopenia risk in inpatient frail older people: a cross-sectional study

**DOI:** 10.1007/s11739-025-04109-9

**Published:** 2025-09-19

**Authors:** Laisa Socorro Briongos Figuero, Miriam Gabella Martín, Fernando Gil Díez, Graciela López Muñiz, Julia Pérez Nieto, Victoria Olivet de la Fuente, Jesús Franco Rodríguez, Ainhoa Martín Galán, Luis Corral Gudino, José Pablo Miramontes González

**Affiliations:** 1 Department of Medicine, Dermatology and Toxicology. Faculty of Medicine, University of Valladolid, Avda. Ramón y Cajal, 7, 47005 Valladolid, Spain; 2 Internal Medicine Service, Santos Reyes Hospital, Avda. Ruperta Baraya 6, 09400 Aranda de Duero, Spain; 3 Internal Medicine Service, Rio Hortega University Hospital, C/Dulzaina 2, 47012 Valladolid, Spain; 4 Pneumology Service, Rio Hortega University Hospital, C/Dulzaina 2, 47012 Valladolid, Spain; 5 Nursing Care Department, Rio Hortega University Hospital, C/Dulzaina 2, 47012 Valladolid, Spain

**Keywords:** Point-of-care ultrasound, Sarcopenia, Multimorbidity, Frailty, Q quality of life, Screening, Nutrition, Active aging

## Abstract

Bedside ultrasound (POCUS) has emerged as a non-invasive, reliable, and practical tool for evaluating muscle mass since sarcopenia poses a significant threat to multimorbidity older people. The aim of the study was to evaluate sarcopenia risk among frail older inpatients using POCUS on rectus femoris muscle (RFM) and explore the relationship between these condition and clinical outcomes. An observational study was conducted including inpatients over 80 years old, admitted due to dyspnea during the winter–spring of 2024. POCUS was done with a 5-MHz convex probe device in RFM (middle point). We determined sarcopenia risk (SARC-F tool), FRAIL scale, handgrip strength, calf circumference (CC), body mass index (BMI), Charlson index (CI), nutritional status (MNA-SF), serum albumin, dependency degree (Barthel index), and quality of life with EQ5D5L questionnaire. Data were analyzed using SPSS v.25.0. The study was approved by the ethics committee. Ultrasound sarcopenia was found in 33% of all participants. POCUS RFM muscle thickness showed moderate positive significant correlation with BMI (*r* = 0.32; *p* = 0.01), arm circumference (*r* = 0.38; *p* = 0.003), CC (*r* = 0.5; *p* < 0.001), and handgrip strength (*r* = 0.25; *p* = 0.04). Otherwise, POCUS RFM muscle thickness showed moderate or weak negative significant correlation with age (*r* = − 0.22; *p* = 0.05), CI (*r* = − 0.24; *p* = 0.01), dependence Barthel index (*r* = − 0.12; *p* = 0.05), nutritional status (*r* = − 0.18; *p* = 0.01), and frailty scale (*r* = − 0.19; *p* = 0.05). Integration of POCUS in comprehensive assessment of quality of life, sarcopenia, nutritional status, and frailty in the oldest old represents a promising approach. This study provides a foundation for implementation of routine sarcopenia screening and intervention programs in the clinical management of multimorbidity in older patients.

**Supplementary Information:**

The online version contains supplementary material available at 10.1007/s11739-025-04109-9.

## Introduction

The progressive aging of the world’s population increases the risk of chronic diseases, although many of our older people experience active aging with high levels of independence, functional capacity, and well-being, despite suffering from one or more diseases. On the other hand, in another group of older patients, there is a decline in health and quality of life, and the development of disabilities, dependence, need for care, and consumption of health resources [1]. These events have resulted in the age of patients admitted to the Internal Medicine Services increasing significantly and this increase is greater than expected due to the general aging of the population [2]. The proportion of extremely older people in Europe has been increasing since the 1990 s, with the life expectancy of the Spanish population being one of the longest in the world [3]. According to the latest national data available, Internal Medicine Services are the ones with the highest number of hospitalizations (19.33%) [4] with the symptom of dyspnea (secondary to heart failure, pulmonary edema, respiratory failure and chronic obstructive lung disease), generating the greatest assistance burden [5].

Fragility is a continuous geriatric syndrome between the healthy and vulnerable older adult, prior to the emergence of functional dependence. It is defined as the decrease in the body's ability to respond to external stressors, causing the individual's risk of falls, functional decline, disability, dependence, institutionalization, and even death [6]. Frail individuals consistently report lower QoL scores and face higher risks of hospitalization, institutionalization, and mortality. Sarcopenia is a geriatric syndrome first coined in 1989 [7]. Recently, the European Working Group on Sarcopenia in Older People (EWGSOP) proposed a clinical–practical definition and developed consensus diagnostic criteria. Thus, sarcopenia is defined as a syndrome of multifactorial etiology characterized by a gradual and widespread loss of skeletal muscle mass, along with poor muscle function (less muscle strength or lower physical performance), which entails a high risk of disability, but also increases the risk of adverse outcomes such as falls, fractures, and loss of independence, loss of QoL, and increased morbimortality, constituting today a geriatric syndrome with great importance [8, 9]. Nutritional status plays a crucial role in this paradigm. Malnutrition, often observed in older population, exacerbates sarcopenia and accelerates the progression toward frailty. The frequency of sarcopenia in our country, taking the EWGSOP2 criteria, is around 33% in women and 10% in men not institutionalized and without functional deterioration, figures that almost double in residents of social healthcare centers [10, 11].

EWGSOP2 sarcopenia criteria include anthropometric evaluations, image tests such as dual X-ray absorption, use of bioimpedance, and dynamic tests like SPPB or Physical Performance Short Battery. This evaluation is not always possible due to technical and patient limitations. Studies evaluating ultrasound as a modality that could be used at point of referral are being investigated for various populations, including those associated with nutritional concern, COPD, and DM type II [12, 13], and a growing body of literature suggests that ultrasonography could be a viable, quick diachronic, and indeed accurate tool to assess muscle mass and quality [8–11, 14–16]. This is where in recent years the use of clinical ultrasound on the bed (known as POCUS in English—point-of-care ultrasounds) has been implemented, being an inexpensive, safe, and reliable method that can be performed by the doctor himself, establishing recently the ultrasound cutting points for this pathology [17, 18]. Ultrasound offers added utilities beyond muscle mass measurement by allowing for the assessment of muscle quality, which is also key to understanding clinical implications in sarcopenia. Its advantages include its lower cost and portability in comparison with other imaging techniques and that it is capable of assessing sarcopenia and its progression by estimating muscle thickness, pennation angle, echogenicity parameters. In this line, studies have demonstrated the potential of ultrasound imaging assessment to diagnose sarcopenia by examining errors in muscle health as regard rectus femoris and tibialis anterior decreased energy efficiency non-invasively with practical methods accompanied a new approach advance for healthcare providers like physical therapists. In patients with the risk of malnutrition, accessibility confers to ultrasound a great potential for the routine screening of sarcopenia in several populations, such as hospitalized older individuals [19, 20]. More recent analysis includes patient care applications analyzing serial muscle loss over time and change in functional capacity using a minimally invasive technique [14, 21–24]. The use of ultrasound in combination with other clinical parameters such as handgrip strength, SARC-F score, nutritional status, and bioimpedance analysis enhances the accuracy of sarcopenia diagnosis.

In geriatric medicine, the complex interrelationships between the quality of life (QoL), sarcopenia, nutritional status, and frailty are of paramount importance, particularly in the oldest people (aged 80 and above). These factors form an intricate web of cause and effect, significantly impacting the health outcomes and overall well-being of this vulnerable population. The aim of this research is to evaluate the usefulness of point-of-care ultrasound in the rapid assessment of sarcopenia risk among inpatient older individuals and associated risk, potentially revolutionizing care for this vulnerable population.

## Methods

The meticulous design described below ensured the homogeneity of the sample, strengthening the internal validity of the study. A holistic approach guaranteed the ability to explore relevant associations and trends, enhancing the external validity of the study.

### Study design

We carried out an observational, cross-sectional, monocentric study for the functional evaluation of our patients and the association between fragility, dependence, risk of malnutrition, ultrasound sarcopenia measures, and health-related quality of life, enrolling consecutive multimorbid older patients, over 80 years old admitted due to dyspnea as a guiding symptom, from February to May 2024 in our medical services (Internal Medicine and Pneumology) of Rio Hortega University Hospital, a third-level hospital. Those with conditions that could independently affect muscle mass like being fed exclusively by enteral or parenteral nutrition, terminal illness that would grant a life expectancy of less than 6 months (evaluated by the PALIAR index for advanced chronic medical conditions [25]), or those unable to provide informed consent by themselves or by legal representation were excluded as well as those discharged from other wards because of intra-hospital transfer.

All included patients had an initial medical standard evaluation, including collection of clinical data, physical examination, and complementary test, so diagnosis management and treatment were established according to clinical protocols. After that, they were classified, based on FRAIL scale results, as having a high probability of frailty (if score of 1 or more) or not, following recent national consensus recommendations [26].

The study was approved by the Ethics Committee for Research with Medicines (CEIm) of the Valladolid West Health Area (Ref. CEIm: 23-PI052). All subjects or their legal representatives gave written consent and our study protocol received approval by the local ethics committee according to the principles of the Declaration of Helsinki.

### Ultrasound measurements

POCUS was performed within 48 h of hospital admission using a portable ultrasound (US) device (MyLabSigma, Esatote, Genova, Italy) carrying three US probes: linear (3–12 MHz), phased array (cardio, 1–5 MHz) and convex (1–5 MHz). POCUS operators were experts in this technique with a probe results variability of 0.5% between them. US examinations were conducted with both convex and linear probes device in the rectus femoris muscle—RFM (middle point). We measured muscle thickness and cross-sectional area (CSA) of the anterior femoral rectum in B-mode, measuring the distance between the anterior and posterior fascia of the muscle, in the middle point between the anterior–superior iliac spine and the upper edge of the patella according to current standards. Muscle measure included the muscle belly and fascia and excluded subcutaneous adipose tissue and skin. Cross-sectional measurements allow for a detailed characterization of muscle mass, aligning with the recommendations of the European Working Group on Sarcopenia in Older People (EWGSOP2) [11, 14]. Patients were positioned supine in 30º-upper body elevation, with legs extended and muscles relaxed. A copious amount of gel was applied to minimize tissue compression and all measurements were made using on-screen calipers at the bedside. The average of three measurements was recorded for each patient. All ultrasound examinations were conducted by four highly experienced and qualified medical doctors according to the standards of Spanish Society of Internal Medicine and European Federation of Internal Medicine criteria. The ultrasound findings were recorded and independently documented by each examiner on each case report forms. RFM normal values were defined according to published data cutoff points were set to 0.7 cm and 0.9 cm for females and males, respectively, while cutoff value of the RFM cross-sectional area (CSA) still remained undefined [11, 14, 17, 18, 22, 27].

### Anthropometric measurements and body composition

Anthropometric measurements were carried out by nursing staff including weight, height, and arm and calf circumferences (CC) (cm) of the patients. To define the reduction in muscle mass, the diameter of the forearm under 22.5 cm or CC under 31 was used as the reference value in our population [28]. Body mass index (BMI) was calculated dividing the weight in kilograms by the square of height in meters. Handgrip strength was measured using a Jamar hydraulic dynamometer manufactured by Talexco [29]. Patients performed three trials with their dominant hand, and the highest value was recorded. EWGSOP2 group recommends that the mean reference for low muscle strength cutoff value is < 30 kg in men and < 20 kg in women [11, 30].

### Nutritional status

Evaluation of the nutritional status was carried out by nursing staff using Mini Nutritional Assessment Short Form (MNA*-*SF), a six question form that identifies older individuals as well nourished (12 points or more: normal, not at risk) or at risk of malnutrition (11 points or below: possible malnutrition), so that the full MNA was needed only if a patient is classified as at risk [31, 32].

Also blood sampling tests were performed in fasting conditions and the analytical variables serum albumin (g/dl), hemoglobin (g/dl), glomerular filtration rate (GFR) (mL/min/1.73m2) (CKD-EPI formula), and total leukocyte (1000/ul) were assessed using an auto-analyzer (Roche Diagnostic, Basel, Switzerland).

### Functional status

Sarcopenia screening risk was evaluated with SARC-F score, a self-reported screening tool that can identify rapidly sarcopenic patients, which include deficiencies in strength, assistance in walking, rising from a chair, climbing stairs, and experiencing falls. The scores range from 0 to 10, and a score equal to or greater than 4 is predictive of sarcopenia and poor outcome [33].

Frailty was assessed using FRAIL scale (Fatigue, Resistance, Aerobic, Illnesses and Loss of Weight), a rapid simple test of five questions relating to five domains: fatigue, resistance, deambulation, comorbidity, and weight lowhich that can be completed in a few minutes. The score ranges from 0 to 5. If the result is equal to or greater than 3 it is considered frail, 1 or 2 defines a patient as pre-frail (1–2), and 0 as robust health status [6, 34]. In our country, a recent national consensus [26] endorsed by Spain Ministry of Health of [35] has shown that a cutoff score of ≥ 3 has low sensitivity for detecting frailty in our population, and that a FRAIL scale score ≥ 1 had a sensitivity of 83.3%, so the recommendation is consider high probability of frailty if score ≥ 1.

EuroQol-5D-5L generic questionnaire was used to evaluathHealth-related quality of life (HRQoL) variables. This tool collects information on five dimensions affecting patient health: mobility, self-care, usual activities, pain/discomfort, and anxiety/depression. In this questionnaire, each dimension has five levels (no problems, slight problems, moderate problems, severe problems, and extreme problems). The combination of the values of all the dimensions generates five-digit numbers that describes the patient’s health state and can be calculated through cross-walk index values calculator by van Hout et al*.* [36]. A visual analog scale (VAS) is the second part of the questionnaire, a 20 cm vertical scale that generates a self-rating of HRQoL. The VAS score ranges from 0 (bad) to 100 (good) [36–39].

### Clinical and demographic variables

Clinical variables and sociodemographic and epidemiological data were obtained from digital medical records. Variables such as age, sex, discharge diagnosis, main comorbidities, length of stay, early 30-day readmission rate, and mortality rate duringt hospital stay were recorded.

Comorbidity was evaluated using Charlson index (CI), which consists of 19 items corresponding to comorbid conditions, which, due to their severity, may increase mortality. Each item is assigned a score and the sum of all item scores is a predictor of mortality: 0–1 point signifies no comorbidity, 2 points low comorbidity and > 3 points high comorbidity. This provides a prediction of the mortality rate (short-term follow-up < 3 years) of 0 (12% mortality/year); 1–2 (26% mortality/year); 3–4 (52% mortality/year) and > 5 (85% mortality/year) [40].

Variables relating to the degree of dependence were determined by nursing staff using Barthel index, a ten-item scale for physical function and ability to complete activities of daily living (ADL) such as feeding, bathing, continence, mobility, and dressing, ranging from 0 to 100, with 100 being the most independent level of function [41].

### Statistical analysis

Sample size was calculated based on the number of mean admissions at medical services in previous years during the same period in which the study was to be carried out. Based on this data, we used GRANMO tool (https://www.datarus.eu/aplicaciones/granmo/) to determine the sample size, after assuming an Error margin of 5% and a confidence level of 95%. A sample size of 42 patients was established for each group to find significant differences between patients with and without frailty.

Quantitative variables were described as average ± standard deviation—SD- (normal distribution) or median and interquartile range (non-normal distribution). Qualitative variables were expressed in frequencies and percentages. HRQoL analysis was carried out according to the instructions of the original authors. The normality of the variables was determined using the Kolmogorov–-Smirnov and Shapiro–Wilk tests. Chi-square test was used for categorical variables and the Student’s T-test and ANOVA for continuous variables. Non-parametric tests were used if the variables did not meet the necessary conditions. Bivariate and multivariate analysis was carried out to assess and analyze statistical associations and risk factors. Pearson correlation and Kruskal–Wallis tests were used to establish the relationship between sarcopenia (defined by POCUS) and other variables (frailty, CI, HRQoL, nutritional status, dependence degree, handgrip strength…). Multivariate analysis was performed and taking into account the sample size and significant variables in bivariate and correlation analysis, the number of variables included in the final model was 6.

Data were analyzed using IBM SPSS Statistics v.25.0 (licensed by the University of Valladolid) and the statistical significance level was set at 0.05.

## Results

### Subject characteristics

Our study enrolled sequentially 107 participants, mean age 92.2 ± 8.5 years. Among the 107 individuals (58.0% male), 59.0% (*N* = 63) were classified as frail, 57.0% (*N* = 61) showed high risk of sarcopenia, 25.0% (*N* = 27) were severely dependent, and the estimated HRQoL was 0.5235 ± 0.450 on a scale between −0.654 and 1 for value set and 53.3 ± 17.0 for visual analog scale (VAS). Corrected CI mean score was 6.4 ± 2.0, and 47.7% (*N* = 51) of patients had high comorbidity (higher in men than in women: 58.0% vs 33.0%, *p* = 0.011). The most common comorbidities were hypertension (75.7%, *N* = 81), chronic respiratory disease (37.4%, *N* = 40), congestive heart failure (37.4%, *N* = 40), atrial fibrillation (33.6%, *N* = 36), type 2 diabetes mellitus (25.0%, *N* = 27), and dementia (12.0%, *N* = 13). Regarding the final diagnosis at the time of discharge, we registered acute lower respiratory infections (42.04%, *N* = 45), acute heart failure (28.04%, *N* = 30), acute exacerbation of chronic obstructive pulmonary disease (13.1%, *N* = 14), flu (9.34%, *N* = 10), lung cancer (4.67%, *N* = 5), and acute pulmonary embolism (2.81%, *N* = 3). Global 30-day readmission rate was 17.8%. Subject clinical and demographic characteristics are shown in Table 1.

**Table 1:** Subject characteristics based on frailty

Characteristics	Not frail (*N* = 44)	Frail (*N* = 63)	*p*-value
Age (years; mean ± SD)	90 ± 8.5	93 ± 8.4	0.150
Biological sex (men)	66.0% (29)	52.4% (33)	0.160
BMI category (obesity)	38.0% (17)	63.5% (40)	0.002*
High sarcopenia risk (SARC-F index)	16.0% (7)	86.0% (54)	< 0.001*
EQoL5D5L value set (mean ± SD)	0.7917 ± 0.24	0.3361 ± 0.48	< 0.001*
EQoL5D5L VAS (mean ± SD)	64.2 ± 15.0	45.8 ± 15.0	< 0.001*
High comorbidity (CI)	43.0% (19)	51.0% (32)	0.280
Short-term mortality rate (52% mortality/year or more)*	43.0% (19)	50.8% (32)	0.220
Severe dependence (Barthel score)	6.8% (3)	38.0% (24)	< 0.001*
MNA-SF test (malnutrition)	2.3% (1)	25.4% (16)	< 0.001*
Albumin (g/dl; mean ± SD)	3.5 ± 0.6	3.15 ± 0.5	0.004*
Leukocyte (1000/ul; mean ± SD)	8.7 ± 3.2	10.1 ± 4.3	0.070
Hemoglobin (g/dl; mean ± SD)	12.5 ± 1.7	12.1 ± 1.8	0.160
GFR (mL/min/1.73m2; mean ± SD)	61.0 ± 21.5	58.2 ± 22	0.500
Hospital stay (days; mean ± SD)	8.1 ± 6	9.1 ± 6.5	0.200
30-days readmission (yes)	16.0% (7)	19.0% (12)	0.440
Mortality rate**	0.0% (0)	8.0% (5)	0.010*

*SD* standard deviation, *BMI* body mass index, *VAS* visual analog scale, *CI* Charlson index, *MNA-SF* Mini Nutritional Assessment Short Form, *GFR* glomerular filtration rate, *CI questionnaire prediction

*Significance values

**During hospital stay

### POCUS and sarcopenia-related data

Regarding sarcopenia, anthropometric measurements and POCUS, both global data and stratified by frailty, are shown in Table 2.

**Table 2:** POCUS and sarcopenia-related data according to Frail scale classification

Measure	All subjects (*N* = 107)	FRAIL scale		
		Not frail (*N* = 44)	Frail (*N* = 63)	*p*-value
BMI (kg/m^2^; mean ± SD)	26.3 ± 6.0	25.5 ± 5.2	27 ± 6.5	0.080
Arm circumferences (cm; mean ± SD)	26.5 ± 5.0	24.7 ± 3.6	27 ± 5.0	0.100
Calf circumferences (cm; mean ± SD)	32.5 ± 6.8	32.8 ± 5.5	32.5 ± 7.2	0.200
Handgrip strength (kg; mean ± SD)	11.6 ± 10.3	15.2 ± 6.8	10.5 ± 9.8	0.040*
POCUS^3^ RFM4 muscle thickness (cm; mean ± SD)	0.9 ± 0.3	0.8 ± 0.15	0.9 ± 0.3	0.010*
POCUS CSA^5^ (cm^2^; mean ± SD)	3.7 ± 1.8	3.3 ± 2.0	3.8 ± 1.8	0.400

*BMI* body mass index, *SD* standard deviation, *RFM* rectus femoris muscle, *CSA* RFM cross-sectional area

*Significance values

Ultrasound sarcopenia measured by RFM muscle thickness was found in 33.0% (*N* = 35) of all participants (taking into account biological sex-based cutoff). These patients had a significantly higher mortality rate during hospital stay (12.5% vs 3.0%; *p* = 0.040), longer hospital stay (8.9 ± 4.0 vs 6.1 ± 2.4; *p* = 0.050), higher Barthel dependence rate (62.5% vs 31.0%; *p* = 0.050), higher frailty prevalence (81.3% vs 65.0%; *p* = 0.060), higher malnutrition risk (31.0% vs 12.0%; *p* = 0.020), lower handgrip strength (7.9 ± 6.5 vs 13.2 ± 5; *p* = 0.050), lower CC (29.2 ± 5.7 vs 34 ± 6.8; *p* = 0.010), and lower EQoL5D5L VAS (42.5 ± 12.0 vs 52.0 ± 7.0; *p* = 0.020), without differences in biological sex, BMI category, 30-day readmission rate, and CI or EQoL5D5L value set.

**Fig. 1:**
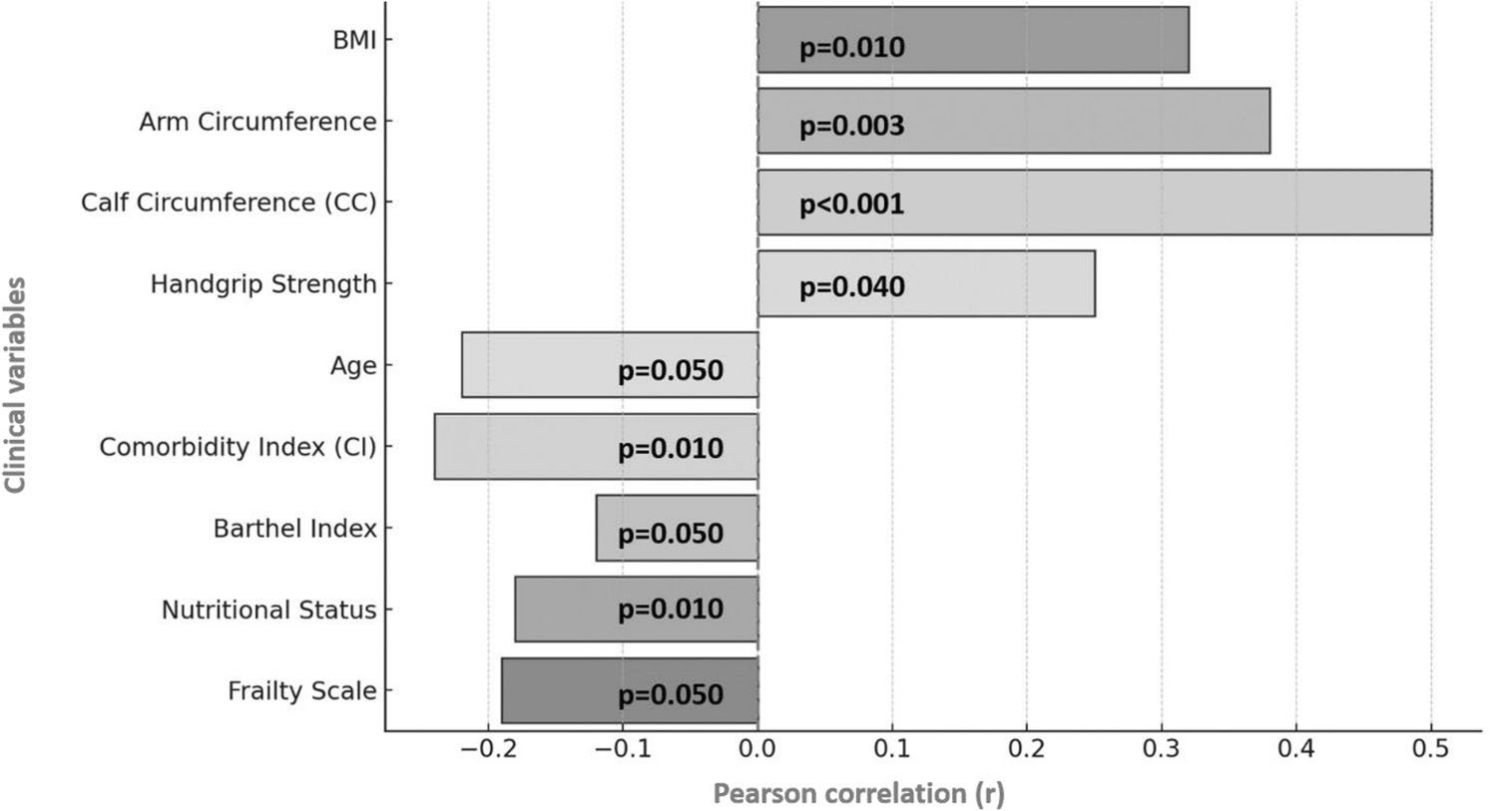
Significant correlations between POCUS RFM muscle thickness and clinical variables

POCUS RFM muscle thickness showed a moderate positive significant correlation with BMI (*r* = 0.32; *p* = 0.010), arm circumference (*r* = 0.38; *p* = 0.003), CC (*r* = 0.50; *p* < 0.001), and handgrip strength (*r* = 0.25; *p* = 0.040). Otherwise, POCUS RFM muscle thickness showed a moderate or weak negative significant correlation with age (*r* = − 0.22; *p* = 0.050), CI (*r* = −0.24; *p* = 0.010), dependence Barthel index (*r* = − 0.12; *p* = 0.050), nutritional status (*r* = − 0.18; *p* = 0.010), and frailty scale (*r* = − 0.19; *p* = 0.050). Correlations were weaker and not significant with SARC-F, leukocytes, serum albumin, length of stay, EQoL5D5L VAS, and EQoL5D5L value set. A global visual representation of significant correlations is shown in Fig. 1.

Multivariate analysis did not identify significant associations or predictive variables, after adjusting for age, biological sex, and comorbidities.

## Discussion

The findings show that sarcopenic and frail patients experience longer hospital stays, higher readmission rates, and increased mortality, underlining the critical importance of early detection and management of these conditions to improve patient outcomes. Surprisingly, we did not find association between POCUS RFM muscle thickness and HRQoL, and serum albumin and SARC-F, maybe due to high comorbidity observed in our patients, higher than that of recent cohorts with similar characteristics [42, 43].

Frailty and sarcopenia are closely linked, with both conditions contributing to poorer outcomes, longer hospital stays, and higher mortality rates, as reported in previous studies [21, 44–46]. In a study involving critically ill older patients, a moderate negative correlation was found between the thickness of the RFM and frailty (*r* = − 0.41; *p* = 0.036), suggesting that muscle ultrasound can be a useful tool for assessing frailty and sarcopenia and predicting clinical outcomes such as weaning success from mechanical ventilation [47].

Our findings align with previous studies and demonstrate that frailty is associated with RFM muscle thickness in older inpatients, regardless of the severity of the disease. The strong association between low muscle mass, as measured by POCUS RFM, and poor clinical outcomes suggests that early identification and management of sarcopenia and frailty could be critical in improving the prognosis for older, multimorbid patients. Interventions such as nutritional support and pharmacological treatments targeting muscle anabolism should be considered to mitigate the adverse effects of both, sarcopenia and frailty. The complexity of the studied population can influence the difficulty in detecting predictor variables in multivariate analysis.

**Fig. 2:**
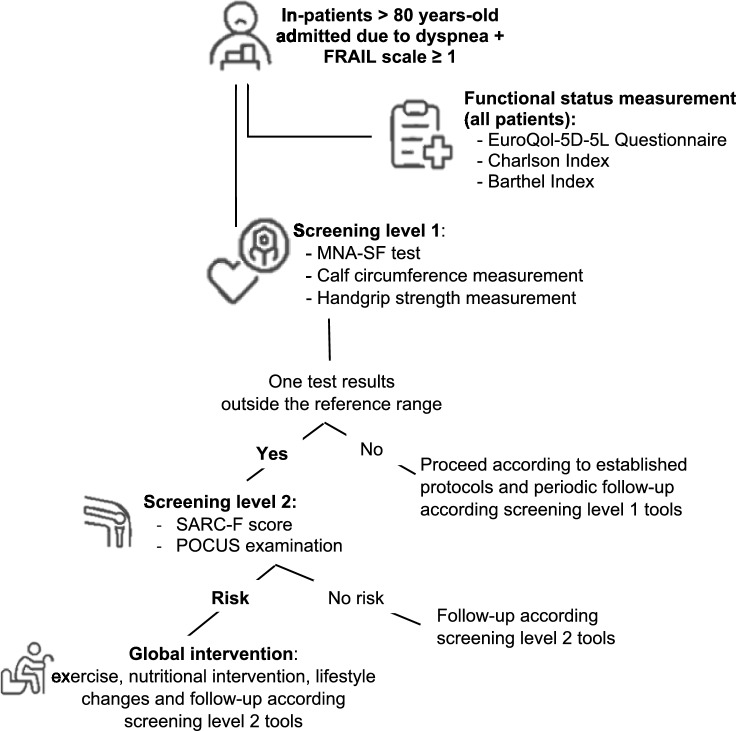
Proposed algorithm for sarcopenia screening in older people. *MNA-SF* Mini Nutritional Assessment Short Form, *POCUS* point-of-care ultrasound. Source: Compilation based on data

Based on the described findings, we propose an algorithm to implement and improve sarcopenia screening in older people through nurse staff and medical team collaboration (Fig. 2). This algorithm promises to be a fast, useful, and non-invasive real-time assessment tool, aimed at improving early detection and, therefore, the quality of life of patients.

### Strengths, limitations, and future research

This study has a number of strengths and several limitations. First of all, as the utility of ultrasound for the assessment of sarcopenia is still a continuously growing field, our investigation allowed us to provide evidence of usefulness of bedside ultrasound to evaluate sarcopenia in older multimorbidity inpatients, a novelty which is limited in literature. Our study also provides useful information about frailty and HRQoL in this population.

Some study limitations are noteworthy. Although the proportion of older people is increasing rapidly throughout the world, especially in Europe, and the aging process and longevity involve increased healthcare resource consumptions, the major limitation may be the very advanced age of the population studied and the dependence degree. Also, the observational design precludes causal inferences. The sample size being monocentric, while adequate, may lower the generalizability of this finding for other populations and may underestimate sarcopenia risk. Additionally, the use of ultrasound, while advantageous, requires operators skilled in its use and can be subject to inter-operator variability and operator dependency. Hypothetically, measuring muscle dysfunction with only grip strength could miss data related to the strength of the leg muscles. Future studies should include larger and more heterogeneous populations and other variables such as changes in echo intensity pattern and echotexture of muscles, complete muscle function test like chair stand test (chair rise test), gait speed test, or short physical performance battery. This would be favorable to investigate in future research important information on the health and function of the muscle, with a view to assessing the effectiveness of specific targeted interventions in reducing sarcopenia- and frailty-related burden. Furthermore, our group designed a multicenter study to confirm the results shown in this pilot study, conducted prospective validation of the proposed algorithm, and found new markers of ultrasound sarcopenia risk in a wide-aged population that allowed us to develop a mobile application aimed at implementing rapid global high accuracy intervention in reversing the situation. Data from future research will help develop new routine frailty and sarcopenia assessment protocols for the management of older patients.

## Conclusion

Determining the causes of disability in geriatric population and preventing its functional deterioration is essential to promote active aging in our older adults, thus avoiding ageism. The intricate interplay of sarcopenia and frailty among older patients grappling with multimorbidities poses a pivotal challenge in contemporary clinical contexts, necessitating a discerning exploration. Early detection of sarcopenia improves the prognosis and quality of life and reduces hospitalization and mortality and, on the other hand, the implementation of nutritional intervention programs in fragile patients highlights the importance of the use of protein supplements to improve muscle mass and its functionality in this type of patients, which could represent a new path of research in this field.

This study provides preliminary evidence of the value of bedside ultrasound in the assessment of sarcopenia and fragility in hospitalized multimorbidity frail older patients, reducing hospitalization and mortality. Point-of-care ultrasound is a promising tool for the rapid and cost-effective method for assessment of sarcopenia risk, frailty, and muscle strength among older inpatients. These are directly related to muscle mass and handgrip strength, making it useful for diagnosing sarcopenia and assessing nutritional status as well. These findings suggest that ultrasound imaging presents a unique potential in terms of revolutionizing the approach of sarcopenia assessment and monitoring by an early identification of muscle dysfunction without radiation exposure by adopting non-invasive POCUS diagnosis. This fact could have significant implications for clinical practice; so, healthcare providers can enhance their ability to identify individuals at risk of sarcopenia and tailor more effective interventions to improve muscle health and overall well-being. Addressing sarcopenia, optimizing nutritional status and quality of life, and mitigating frailty represent key strategies. However, further studies focusing on developing integrated, personalized approaches and establishing normative data specific to the older population are needed to establish clear guidelines for their implementation in clinical practice.

## Supplementary Information

Below is the link to the electronic supplementary material.

Supplementary file1 (DOCX 13 KB)

## Data Availability

The data that support our findings are available from the corresponding author upon reasonable request. The data are not publicly available due to privacy or ethical restrictions. The principal investigator of this study had full access to all the data and assumes responsibility for the accuracy of the data analysis.
