# Supplementary material for: Usefulness of point-of-care ultrasound for rapid assessment of sarcopenia risk in inpatient frail older people: a cross-sectional study
Source: Intern Emerg Med. 2025 Sep 19;20(8):2379–88. doi: 10.1007/s11739-025-04109-9 (PMC12672653; doi:10.1007/s11739-025-04109-9)
Supplement: Supplementary file 1 — Supplementary file1 (DOCX 13 KB) [file 11739_2025_4109_MOESM1_ESM.docx]

**SUPPLEMENTARY DATA. SCALES**

FRAIL Scale.

FRAIL scale (Fatigue, Resistance, Aerobic, Illnesses and Loss of Weight) is a rapid simple test of 5 questions relating to 5 domains: fatigue, resistance, deambulation, comorbidity and weight loss, that can be completed in few minutes. The score ranges from 0 to 5. If the result is equal to or greater than 3 it is considered that there is a high probability of frail

1. Fatigue: How often in the past four weeks have your felt more tired than usual?

2. Resistance: By yourself and not using aids, do you have any difficulty walking up 10 steps without resting??

3. Ambulation: Do you have any difficulty walking several hundred yards?

4. Illnesses: Do you have any of the following illnesses: hypertension, diabetes, cancer, chronic lung disease, heart attack, congestive heart failure, angina, asthma, arthritis, stroke, or kidney disease?

5. Loss of weight: Have you lost over 5% of your total body weight in the past?

SARC-F Screen for Sarcopenia.

SARC-F score is a self-reported screening tool that can identify rapidly sarcopenic patients, which include deficiencies in Strength, Assistance in walking, Rising from a chair, Climbing stairs, and experiencing Falls. The scores range from 0 to 10 and a score equal to or greater than 4 is predictive of sarcopenia and poor outcome.

1. Strength: How much difﬁculty do you have in lifting and carrying 10 pounds?

2. Assistance in walking: How much difﬁculty do you have walking across a room?

3. Rise from a chair: How much difﬁculty do you have transferring from a chair or bed?

4. Climb stairs: How much difﬁculty do you have climbing a ﬂight of 10 stairs?

5. Falls: How many times have you fallen in the past year?
